# Pneumocephalus and Meningitis as Complications of Mastoiditis

**DOI:** 10.1155/2019/7876494

**Published:** 2019-02-19

**Authors:** Conor Barry, George Rahmani, Diane Bergin

**Affiliations:** Department of Radiology, Galway University Hospitals, Galway, Ireland

## Abstract

Pneumocephalus in the absence of trauma, tumour, or surgery is a rare entity. We report a case of a 73-year-old lady who presented with sepsis leading to confusion and unresponsiveness. A CT of brain revealed mastoiditis, sinusitis, and associated pneumocephalus. Further investigations led to an eventual diagnosis of pneumococcal meningitis. The combination of pneumocephalus and meningitis as complications of mastoiditis is rare with very few cases published in the literature. We describe one such case.

## 1. Case Report

A seventy-three-year-old lady was brought in by ambulance to the emergency department with increasing confusion. She had a history of type 2 diabetes mellitus and hypertension. The patient had become gradually unwell for three weeks prior to admission, complaining of lethargy, myalgia, and a dry cough. On admission to the emergency department, she was confused with a Glasgow coma scale of 14/15. She was pyrexic (40.6°C), tachycardic (104 BPM), and hypertensive (186/82), with a respiratory rate of 26 and oxygen saturations of 93% on room air. Physical examination yielded coarse crepitations in her left lung base. The rest of the examination was otherwise unremarkable. Of note, there was no ear discharge, nor were there any defects in the tympanic membranes. Initial blood results showed a leucocytosis of 14.4 x 109/L, with a neutrophilia (13.3 x 109/L). Her C reactive protein was raised (295 mg/L) and her blood lactate was elevated (4.9 mmol/L) with an acidosis (pH 7.29). Her ECG showed sinus tachycardia. She had left lower zone consolidation on her chest X-ray. Shortly following admission, she rapidly deteriorated, becoming unresponsive and requiring urgent intubation. Intravenous ceftriaxone and acyclovir were administered and an urgent CT brain was performed prior to lumbar puncture.

**Figure 1:**
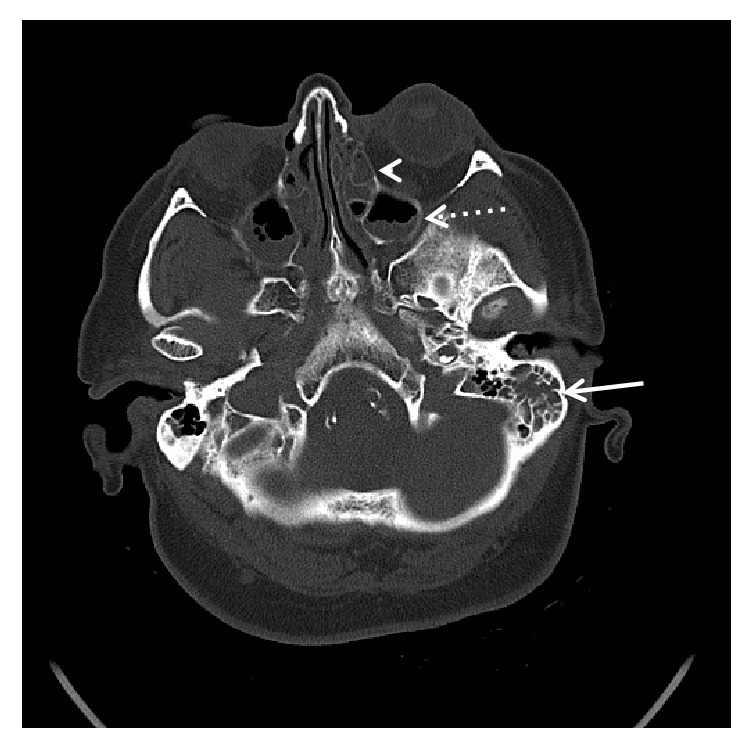
Axial CT demonstrating opacification of the mastoid air cells (solid arrow), ethmoid sinuses (arrowhead), and maxillary sinuses (dashed arrow) in keeping with mastoiditis and sinusitis.

**Figure 2:**
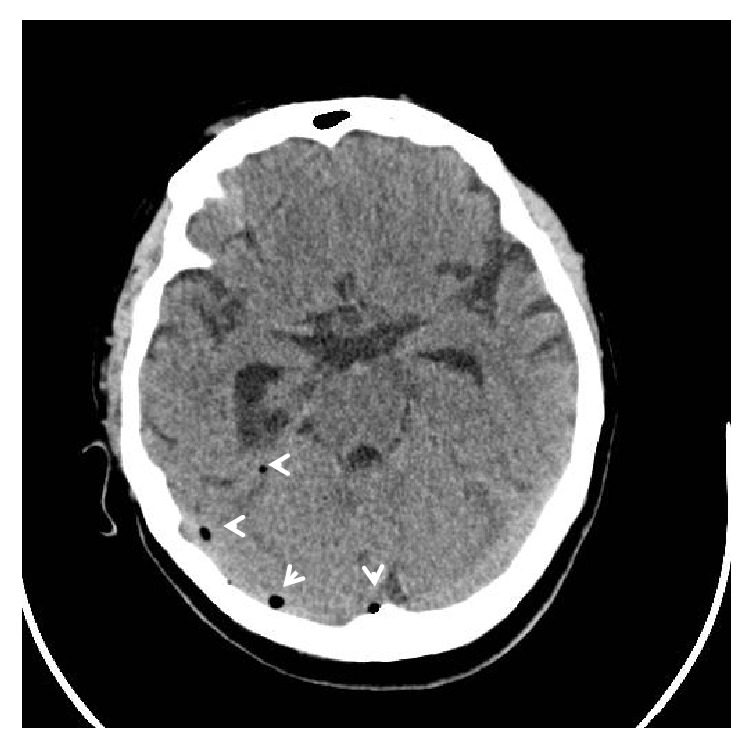
High resolution axial CT showing a focal osseous defect in the posterior mastoid (arrow).

**Figure 3:**
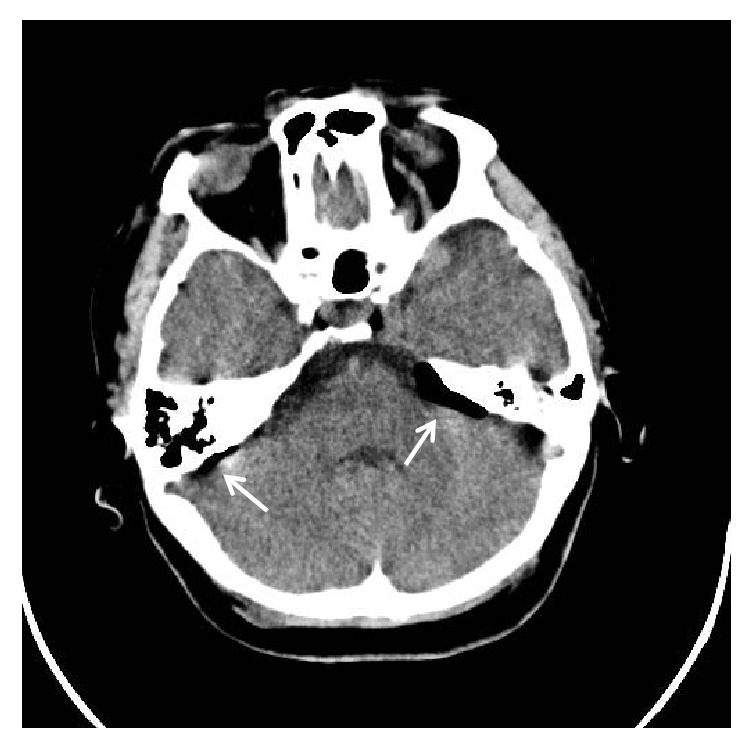
Axial CT brain demonstrating pneumocephalus in the posterior cranial fossa (arrowhead).

**Figure 4:**
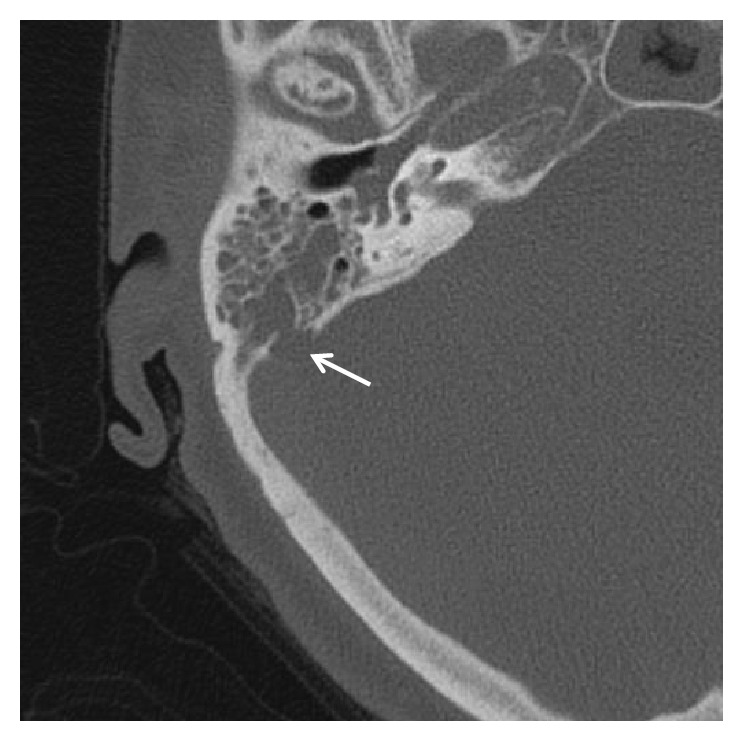
Axial CT brain demonstrating pneumocephalus in the posterior cranial fossa (arrow).

The CT brain showed opacification of the mastoid air cells as well as the ethmoid and maxillary sinuses in keeping with mastoiditis and sinusitis (Figure 1). There was pneumocephalus with extra-axial air in the posterior cranial fossa bilaterally (Figures 2 and 3) and a focal osseous defect in the posterior wall of the right mastoid air cells causing direct communication with the posterior cranial fossa (Figure 4).

A lumbar puncture was then performed with gram-positive cocci seen on gram-staining and 3,200 white cells/cmm. CSF culture yielded growth of* S. pneumoniae* and this was subsequently confirmed with molecular testing for* S. pneumoniae* DNA and a diagnosis of pneumococcal meningitis was made. She was treated with intravenous antibiotics for a total of two weeks and bilateral tympanostomies were performed for management of her mastoiditis. She subsequently improved and made a full and uneventful recovery. A repeat CT brain at the time of discharge showed resolution of her mastoiditis and pneumocephalus.

## 2. Discussion

Pneumocephalus is defined as “air or gas in the cranial cavity” and is, classically, seen in the context of trauma, tumours, postoperative neurosurgical patients, radiation necrosis, or meningitis/encephalitis caused by gas forming organisms [1–3]. Pneumocephalus as a complication of pneumococcal meningitis is extremely rare with only a handful cases reported in the literature [4–9]. Usually pneumocephalus is asymptomatic but signs and symptoms are variable ranging from confusion and altered mental status, to headache, vomiting, and seizures. Occasionally pneumocephalus can cause intracranial hypertension behaving physiologically like a space occupying lesion and potentially leading to brainstem herniation [1, 10]. It has important implications in anaesthesia, and there is at least a theoretical risk of tension pneumocephalus if nitrous oxide is used in the presence of pneumocephalus [11]. This is of particular importance in postoperative neurosurgical patients that can potentially require repeat surgery over a short period of time.

Pneumocephalus is a rare complication of pneumococcal meningitis and has also been reported in the presence of otitis media, sinusitis, and mastoiditis as in this case [3, 4, 6, 8]. The mechanism in this case was most likely due to a cortical defect in the right mastoid as a result of the patient's mastoiditis allowing direct communication with the posterior cranial fossa. Management of pneumocephalus is based on the patient's clinical status, magnitude, and progression of the pneumocephalus and the underlying aetiology. Most cases resolve with conservative management and close observation; however the actual rate at which the air is absorbed is unknown. In general, 75-85% of patients show radiological resolution of air within the first week [11]. Diagnosis is usually made using CT and is sensitive for volumes of air as little as 0.5ml [7]. In the case of an unwell patient with signs of mastoiditis and associated pneumocephalus the radiologist and clinician should consider pneumococcal meningitis in the differential diagnosis and proceed to investigate and treat the patient accordingly.
